# A weighted string kernel for protein fold recognition

**DOI:** 10.1186/s12859-017-1795-5

**Published:** 2017-08-25

**Authors:** Saghi Nojoomi, Patrice Koehl

**Affiliations:** 1 Biotechnology program, University of California, Davis, 1, Shields Avenue, Davis, 95616 CA USA; 2 Department of Computer Science and Genome Center, 1, Shields Avenue, Davis, 95616 CA USA

**Keywords:** String kernel, Protein fold recognition, Amino acid substitution matrices

## Abstract

**Background:**

Alignment-free methods for comparing protein sequences have proved to be viable alternatives to approaches that first rely on an alignment of the sequences to be compared. Much work however need to be done before those methods provide reliable fold recognition for proteins whose sequences share little similarity. We have recently proposed an alignment-free method based on the concept of string kernels, SeqKernel (Nojoomi and Koehl, *BMC Bioinformatics*, 2017, 18:137). In this previous study, we have shown that while Seqkernel performs better than standard alignment-based methods, its applications are potentially limited, because of biases due mostly to sequence length effects.

**Methods:**

In this study, we propose improvements to SeqKernel that follows two directions. First, we developed a weighted version of the kernel, WSeqKernel. Second, we expand the concept of string kernels into a novel framework for deriving information on amino acids from protein sequences.

**Results:**

Using a dataset that only contains remote homologs, we have shown that WSeqKernel performs remarkably well in fold recognition experiments. We have shown that with the appropriate weighting scheme, we can remove the length effects on the kernel values. WSeqKernel, just like any alignment-based sequence comparison method, depends on a substitution matrix. We have shown that this matrix can be optimized so that sequence similarity scores correlate well with structure similarity scores. Starting from no information on amino acid similarity, we have shown that we can derive a scoring matrix that echoes the physico-chemical properties of amino acids.

**Conclusion:**

We have made progress in characterizing and parametrizing string kernels as alignment-based methods for comparing protein sequences, and we have shown that they provide a framework for extracting sequence information from structure.

**Electronic supplementary material:**

The online version of this article (doi:10.1186/s12859-017-1795-5) contains supplementary material, which is available to authorized users.

## Background

Traditional approaches to comparing two protein sequences start with strings of letters, where each letter corresponds to an amino acid type, and a separable scoring function for comparing these letters, to find either the best global alignment [1] or the best local alignment between the two sequences [2]. Unfortunately, it is not easy to find the parameters of a scoring function that best captures the similarity between amino acid types. This has led to the development of many types of scores in the form of substitution matrices in the hope of producing biologically meaningful sequence alignments [3–6]. In addition, when the similarity between the two proteins to be compared is low, the quality of the corresponding sequence alignment is usually lacking. Therefore sequence alignment techniques are usually poor methods for classifying proteins into folds [7] or detecting homology [8, 9], both essential tasks in the hope of solving the protein structure prediction problem. There have been many methods developed to circumvent these problems. More reliable detection of structure similarity can be achieved for example if sequence similarity is defined on the basis of families of sequences, rather than on the basis of the native sequence alone. This fact is at the root of all profile methods used in modern database searching programs such as PSIBLAST [10] and HMMER [11]. Those methods still rely on the concept of alignments, with all its limitations that we discuss below.

It is interesting to note that the scoring schemes associated with sequence alignment methods consider individual amino acids only and not directly oligomers. One option to improve upon these methods involves considering multiple amino acids at once. This idea has led to the concept of “alignment-free” methods which have been developed over the past three decades (for review, see [12–14]). Alignment-free methods rely on the frequencies of words of a fixed length, *k*, also denoted as k-mers. Once the frequency distribution functions of such k-mers have been computed for two sequences, the distance between those two sequences is assimilated to the distances between those distributions, using different definitions of distances [13, 15]. Other implementations are based on word matches of different lengths [16, 17]. It should be noted that all these methods are based on exact word matches. Exact matches, however, are bound to limitations, due to strong correlations between amino acids at neighboring positions. A solution to this limitation was proposed, the so-called *spaced seeds* methods that defines patterns with *match* and possible *don’t care* positions [18–21]. Another class of alignment-free methods for comparing protein sequences that are directly relevant to this work are the string kernel based methods [22–29].

As mentioned above, the sequence alignment methods as well as the recent string kernel methods depend critically on a scoring, or substitution matrix. Those substitution matrices basically encode amino acids as arrays of numerical values, where those values are derived from statistical analyses of reference alignments (the PAM and BLOSUM matrices), or from the physical and chemical properties of amino acids [30–32]. While those matrices have been used in the context of fold recognition problems, they have not been optimized for such a task. There have been attempts to perform such an optimization [33–35]; none, however, have yet surpassed the well accepted BLOSUM62 matrix.

In this paper we describe a new weighted string kernel that attempts to combine the benefits of the local string kernels [27, 28] that use a substitution matrix and of the weighted degree kernels that consider weighted sums of kernels obtained with fixed length k-mers [25]. It is an extension of a preliminary study in which we introduced an unweighted kernel, SeqKernel, and showed its applications to protein fold recognition [29]. In this preliminary study, we have shown that the kernel values computed by SeqKernel show dependencies on sequence length, and that those dependencies can be minimized by changing the values of its parameters. The motivations for this work are twofold. First, we introduce a weighted version of SeqKernel, WSeqKernel, and show that with the proper weighting scheme, the impact of sequence length can be fully eliminated. Second, we use the fact that the string kernel is differentiable with respect to the elements of its input substitution matrix to optimize this matrix such that the kernel scores match with structural scores for pairs of proteins. Starting with the identity matrix, we show that this procedure generates a substitution matrix that recovers the physico-chemical properties of the twenty amino acids.

## Methods

The weighted string kernel considered here, referred to as WSeqKernel, is inspired by the convolution string kernels introduced by D. Haussler [36], the local alignment kernel presented by Saigo et al. [27], and the string kernel of Smale and co-workers [28]. An unweighted version was presented in details in Nojoomi and Koehl [29]. We provide here the key elements of its construction, emphasizing the differences with those kernels. Readers are referred to the original papers for a more detailed presentation, notably for the proofs of the mathematical properties that are relevant to kernels in general.

### The weighted string kernel

**Fig. 1:**
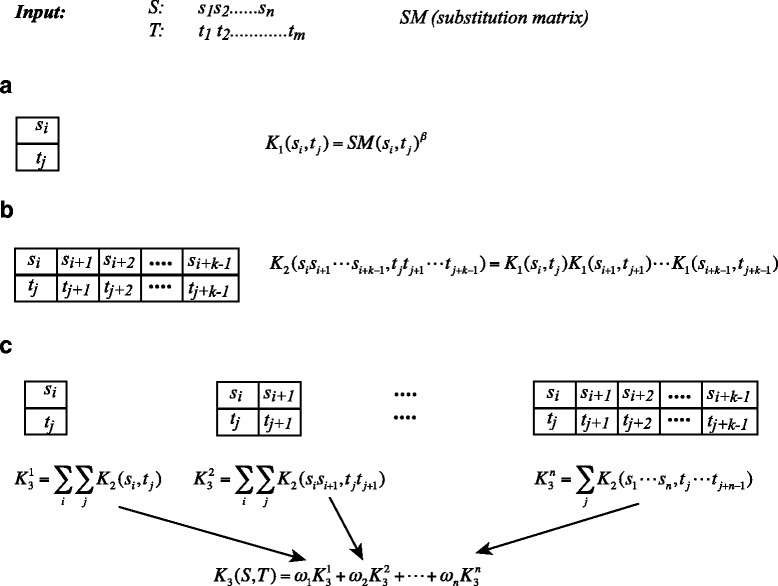
The weighted string kernel WSeqKernel. **a** Comparing two amino acids, **b** Comparing two strings of length *k*, **c** Comparing two sequences

Figure 1 depicts the major steps that define the string kernel, WSeqKernel. The input of WSeqKernel is a pair of sequences, **S**=(*s*_1_,…,*s*_*n*_) and **T**=(*t*_1_,…,*t*_*m*_) of lengths *n* and *m*, respectively, and a substitution matrix denoted *SM*. We note that *n* and *m* may be different; we set *p*= min(*n,m*). Examples of *SM* include the matrices representing the raw data of any BLOSUM matrices [5], namely the raw counts of how often amino acid *i* is substituted by amino acid *j* in a set of selected protein sequence alignments. Such a matrix is normalized such that the sum over each of its row is 1.

We first define a kernel for amino acid pairs, namely a measure of similarity between two amino acids (Fig. 1 a). Given a strictly positive real number *β*, we define the function *K*_1_ as:

1 $$\begin{array}{@{}rcl@{}} K_{1}(s_{i},t_{j}) = SM(s_{i},t_{j})^{\beta}  \end{array} $$

*K*_1_ is a kernel, as long as *SM* is symmetric, positive definite and *β* is strictly positive. The same definition was used in [27, 28].

The second step in the string kernel method implemented in WSeqKernel is to define a kernel for comparing two strings of the same length, *k* (Fig. 1 b). Let *k* be a strictly positive integer and let *S*_*k*_=(*s*_*i*_,…,*s*_*i*+*k*−1_) and *T*_*k*_=(*t*_*j*_,…,*t*_*j*+*k*−1_) be two substrings of *S* and *T*, respectively, both of length *k*. Such substrings are usually referred to as k-mers. It is important to note that a k-mer is a contiguous substring of a sequence, namely that we do not consider gaps. The function $K_{2}^{k}$ defined by:

2 $$\begin{array}{@{}rcl@{}} K_{2}^{k}(\mathbf{S_{k}},\mathbf{T_{k}}) = \prod_{l=1}^{k} K_{1}(s_{i+l-1},t_{j+l-1})  \end{array} $$

is a kernel on the space of strings of length *k*. We note that $K_{2}^{k}$ is a convolution kernel [36].

The kernel between two sequences **S** and **T** with different lengths *n* and *m*, respectively, is then computed by considering all combinations of substrings *S*_*k*_ and *T*_*k*_ is *S* and *T*, for all *k* in [ 1,*p*] (Fig. 1 c). We define

3 $$\begin{array}{@{}rcl@{}} K_{3}^{k}(S,T) = \sum\limits_{S_{k} \in \mathbf{S}} \sum\limits_{T_{k} \in \mathbf{T}} K_{2}^{k}(S_{k},T_{k}) \end{array} $$

where $K_{2}^{k}$ is the kernel on substrings of length *k* define above.

The kernel value for the two sequences **S** and **T** is then computed as a weighted sum of the kernel values $K_{3}^{k}$ for all possible values of *k*:

4 $$\begin{array}{@{}rcl@{}} K_{3}(\mathbf{S},\mathbf{T}) = \sum\limits_{k=1}^{n} \omega(k) K_{3}^{k}(\mathbf{S},\mathbf{T}).  \end{array} $$

where *ω*(*k*) is a positive weight that depends on *k*.

Finally, we define the correlation kernel $\hat {K}_{3}$ as:

5 $$\begin{array}{@{}rcl@{}} \hat{K}_{3}(\mathbf{S},\mathbf{T}) = \frac{K_{3}(\mathbf{S},\mathbf{T})}{ \sqrt{K_{3}(\mathbf{S},\mathbf{S})K_{3}(\mathbf{T},\mathbf{T})}}  \end{array} $$

$\hat {K}_{3}$ is the sequence kernel considered in this paper. Following [28, 29, 36], we make the following remarks:

i) The input kernel matrix *G* is not a traditional substitution matrix, as it does not involve applying the logarithm function on the probability measures. While the latter is needed to make scores additive, a necessary condition to enable the use of dynamic programming algorithms to generate pairwise sequence alignment, it is not needed for the string kernel we use here. Note that this differs from the local alignment kernel that is designed to mimic pairwise alignment.
ii) The kernel *K*_3_ is computed as a weighted sum of the individual kernels $K_{3}^{k}$ that are computed with fixed *k*, akin to the weighted degree kernels [26]. As such, it differs from the string kernel introduced by Smale and colleagues [28] and originally implemented in SeqKernel [29]. Different options for those weights are presented in the result section in the paper.
iii) As defined, $\hat {K}_{3}$ does not consider gap penalties, or even gaps. We consider this as an advantage, as it does reduce the number of parameters.
iv) The string kernel $\hat {K}_{3}$ is a similarity measure in the space of sequences. Notice that for all sequences **S**, $\hat {K}_{3}(\mathbf {S},\mathbf {S}) = 1$. This similarity measure can be transformed into a distance measure, using $D(\mathbf {S},\mathbf {T}) = \sqrt {2-2\hat {K}_{3}(\mathbf {S},\mathbf {T})}$. *D*(**S**,**T**) takes values between 0 and $\sqrt {2}$.

### Implementing the weighted string kernel

The implementation of the weighted string kernel follows closely the implementation of SeqKernel [29]. For completeness, we provide here a flowchart of the algorithm.



The time complexity of this algorithm is *O*(*nmk*_*max*_), which remains large for protein sequence comparison. For two sequences *S* and *T*, the algorithm above is run three times, for the pairs, (**S**,**T**), (**S**,**S**), and (**T**,**T**). The three kernel values are then combined according to Eq. 5 to generate the correlation kernel of the two sequences.

### Optimization of the amino acid kernel *K*_1_

Given a kernel matrix *K*_1_ (see Eq. 1) that defines the similarities of pairs of amino acids, WSeqKernel computes sequence similarity scores that are expected to mimic structure similarity scores. Here we are concerned with the problem of optimizing *K*_1_ such that the similarity between the sequence and structure scores is maximized. To perform this optimization, we need an objective function, derivatives of this objective function with respect to the elements of *K*_1_, as well as a procedure that enforces that *K*_1_ remains symmetric, positive, and definite during the optimization. We discuss the former and latter here, and refer the reader to the Additional file 1 for a complete description of the computation of the derivatives, as well as of the algorithm that implements this procedure.

**Objective function.** Let $\mathcal {L}$ be a set of pairs of proteins. We denote by *N* the cardinality of $\mathcal {L}$. For each pair *n* of proteins in $\mathcal {L}$, we compute the alignment between their structures using STRUCTAL [37], and record its SAS score, *Y*_*n*_. This score is an input of the procedure. In parallel, we compute the kernel between their sequences, using WSeqKernel, and record it as *X*_*n*_. *X*_*i*_ is a non-linear function of the elements of the kernel *K*_1_. Our objective is to optimize the degree of linear dependence between the two variables *Y* and *X*. We quantify this linear dependence using the Pearson’s correlation coefficient, which we denote as *P*:

6 $$\begin{array}{@{}rcl@{}} \begin{aligned} P=\frac { N \sum_{n=1}^{N} Y_{n} X_{n} - {\sum\nolimits}_{n=1}^{N} Y_{n} {\sum\nolimits}_{n=1}^{N} X_{n}} {\sqrt{N {\sum\nolimits}_{n=1}^{N} Y_{n}^{2} - \left({\sum\nolimits}_{n=1}^{N} Y_{n} \right)^{2}} \sqrt{N {\sum\nolimits}_{n=1}^{N} X_{n}^{2} - \left({\sum\nolimits}_{n=1}^{N} X_{n} \right)^{2}} }  \end{aligned} \end{array} $$

The values of *P* are in the range -1 to 1. Note that the SAS score *Y* is akin to a distance measure, while the measure *X* is based on a kernel. As such, large values for *X* are expected to correspond to small values for *Y*, and vice versa. Optimizing the linear dependence between *X* and *Y* is therefore a minimization, i.e. we attempt to push *P* to be as close as possible to -1.

**Maintaining the kernel** ***K***_**1**_ **positive definite.** Direct minimization of the objective function defined by Eq. 6 is likely to fail as there is no guarantee that the matrix *K*_1_ stays positive definite. One option to circumvent this problem is to consider the Cholesky factorization of *K*_1_. Indeed, any positive definitive real matrix *K*_1_ can be decomposed as

7 $$\begin{array}{@{}rcl@{}} K_{1} = L L^{T}  \end{array} $$

where *L* is a lower triangle matrix. Conversely, if *K*_1_ is a matrix that can be written as *LL* ^*T*^ for some invertible lower triangular matrix *L*, then *K*_1_ is positive definite. The latter provides a framework for enforcing positive definiteness, namely we set the parameters of the optimization to be the matrix *L*, the Cholesky factorization of the kernel *K*_1_. We do need to impose that the matrix *L* remains invertible, which is achieved by preventing any of the coefficients *L*(*i,i*) to become zero.

### Datasets

We used in this study the same datasets as in Reference [29]; we describe them here for sake of completeness. Briefly, the first set of structures considered in this study consists of 10619 domains from the CATH [38] v4.0 domains, each with a CATH classification. As we focus on protein fold recognition, we consider the first three levels of CATH, Class, Architecture and Topology, to give a CAT classification. We refer to a set of structures with the same CAT classification as a fold. Using a set of structures with sufficient sequence diversity ensures that the data is duplicate-free and that the problem of detecting structural similarity is non-trivial for all pairs of proteins considered. The 10619 structures were selected as follows: (i) Randomize the list of 235,858 CATH v4.0 domains; (ii) Start with the first domain on the randomized list, and remove from the list all domains that share significant sequence similarity with it (FASTA [39] E-value <10^−4^). (iii) Repeat step (ii) with all domains in the list that have not been removed, until there are no domains left for selection. The set of 10619 domains resulting from this procedure is referred to as CATH40e4.

There are 1363 folds in CATH40e4, many of which only contain a single element (734). To facilitate statistical analysis, we selected five of the most populated folds in CATH40e4 as a more specific test set, including at least one fold from each CATH class: CATH fold 1.10.10, a fully *α* fold (arc repressor, 381 representatives), CATH fold 2.60.40, a fully *β* fold (immunoglobulin-like, 555 representatives), and three alternating *α*/*β* folds: 3.20.20, (TIM-like, 251 representatives), 3.30.70, (two layer sandwich, 368 representatives) and 3.40.50 (Rossmann fold, 1278 representatives). These five folds include a total of 2833 proteins (set CATH2833).

For statistical significance, we generated in parallel a set of ten CATH2833-like datasets by repeating the procedure above, starting with different randomized lists of the CATH4.0 domains. These datasets are referred to as setI, for I between 1 and 10.

### ROC analysis of protein fold recognition

We quantify the effectiveness of a distance measure in identifying correctly if two sequences correspond to proteins that belong to the same CATH class using the receiver operating characteristic (ROC) analysis [40], following the procedure described in Nojoomi and Koehl [29].

### Principal component analysis of a substitution matrix

A substitution matrix *K* can be assimilated to a data matrix in which a set of N “objects” (usually 20 amino acids) are characterized by a set of P measured “features” (the usually 20 scores for substituting one amino acid into another). As such, each amino acid can be considered as a point in a P-dimensional space. Not all P features are equally important, however, and some of these features may be highly correlated. To capture the principal components that describe the amino acids and thereby reduce the dimension of the space in which they lie, it is common to perform a Principal Component Analysis (PCA). PCA can be thought of as fitting an N-dimensional ellipsoid to the matrix *K*, where each axis of the ellipsoid represents a principal component. If some axis is small, then the variance along that axis is also small, and by omitting that axis we lose only a small amount of information. To find the principal components, we first center the values for each feature by subtracting their means:

8 $$\begin{array}{@{}rcl@{}} K_{c}(i,j) = K(i,j) - \frac{1}{N}\sum\limits_{k=1}^{N} K(k,j) \end{array} $$

We then estimate the covariance matrix *C* of the matrix *K* from the centered matrix *K*_*c*_:

9 $$\begin{array}{@{}rcl@{}} C = \frac{1}{N-1} K_{c} K_{c}^{T} \end{array} $$

where a factor N-1 is used instead of N as the mean value of the P features are computed from the matrix *K*, and not from the true distribution. Then, we calculate the eigenvalues of this covariance matrix and their corresponding eigenvectors. The latter provide the directions of the principal components, while the former give the corresponding contribution of that component to the total variance of *K*.

### Reproducibility

We have implemented this procedure into the program WSeqKernel, whose source code is available at the URL http://nook.cs.ucdavis.edu/~koehl/Research/Research_seqanal.html or upon request to the authors. WSeqKernel takes as input two sequences, a substitution matrix *GN*, values for the two parameters *β* and *k*_*max*_, as well as a flag indicating the choice of kernel weighting scheme. It gives as output the value of the correlation kernel $\hat {K}_{3}$ for those two sequences (a similarity measure), as well as the corresponding distance.

## Results

Two proteins with similar sequences almost always share the same structure. The reverse, however, is not always true: Rost [9] has shown that pairs of proteins with similar structures possess, on average only 8–10% sequence identity: this observation is one of the reasons that it is difficult to classify proteins based on sequence information. Here, we test an alternative approach to pairwise sequence comparison. We propose to use a weighted string kernel that provides an alignment-free measure of the similarity of two protein sequences. We use that measure to classify protein sequences and compare the corresponding classification results with classifications derived from 3D structures and sequences. Our aims are twofold. First, we parameterize the weighted string kernel such that it performs better than sequence alignment based methods on fold recognition problems.

Second, we use the weighted string kernel to derive a scoring matrix for amino acid similarities that captures the properties of the structural scores. We use CATH2833 as our test set. CATH2833 is a database of 2833 protein sequences that covers the three main classes of CATH [38]: one fully *α* fold, one fully *β* fold, and three *α*/*β* folds (see “Methods” section). CATH2833 was designed such that the sequences of any pair of proteins in the set have statistically no similarity (FASTA [39] E-value >10^−4^).

### Parameterizing the weighted string kernel

**Fig. 2:**
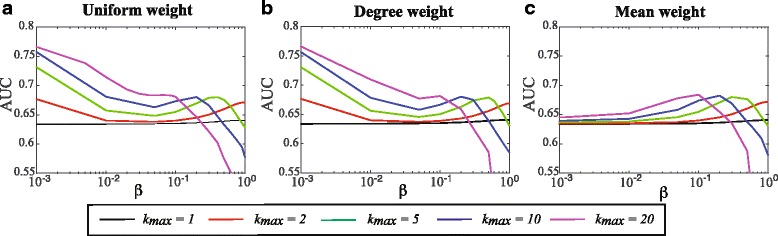
Parameterizing the weighted string kernel WSeqKernel. The string kernel defined in this paper is defined by two parameters, *β* and *k*_*max*_, and a weighting scheme to combine the individual kernels for different k-mer sizes (see text for details). We varied the two parameters in the respective ranges [10^−3^,1] and [1,20]; for each corresponding pairs of values, we applied the corresponding kernel to compute the similarities of all pairs of proteins in CATH2833 and checked the rankings of these similarities with the CATH classification of the proteins, using a ROC analysis. The corresponding AUC values are reported in panels **a**, **b**, and **c** for the three weighting schemes that are compared, respectively. High values of AUC indicate better fold recognition

The weighted string kernel considered in this paper depends on the input substitution matrix *GN*, the weighting scheme for combining the kernels for fixed length k-mers, and two parameters, the power coefficient *β* that is used to compute the Hadamard power of the input substitution matrix *S* (see Eq. 1), and *k*_*max*_, the longest k-mers considered in the comparison of the two sequences. We set the substitution matrix to be BL62, i.e. the raw count kernel matrix derived from the BLOSUM62 matrix (referred to as BLOSUM62-2 in [28]). We consider three possible weighting schemes. In the *uniform weight* scheme, the coefficients *ω*(*k*) are set equal to 1 for all *k*. This corresponds to the unweighted kernel of Smale et al. [28]. In the *degree weight* scheme, the coefficient *ω*(*k*) are set to 2(*k*_*max*_−*k*+1)/(*k*_*max*_(*k*_*max*_+1)), i.e. the weights considered in the weighted degree kernel [25]. Finally, we introduce a new set of weights, *ω*(*k*)=1/((*n*−*k*+1)(*m*−*k*+1)), where *n* and *m* are the lengths of the two sequences *S* and *T* that are compared. Note that there are *n*−*k*+1 and *m*−*k*+1 k-mers in the two sequences *S* and *T*. The latter weight is therefore equivalent to taking the average of the contributions of all k-mers from the two sequences. We refer to this scheme as the *mean weight*. For each weighting scheme, we have tested a range of values for *β* from very small, 10^−3^, to relatively large, 1, and a range of values for *k*_*max*_, from 1 (i.e. single amino acid comparison) to 20. For pairs of values (*β*, *k*_*max*_) taken from their respective ranges, we computed the similarity scores for all pairs of proteins in CATH2833 and assessed the ability of those scores for fold recognition using a ROC analysis (see Methods). The resulting AUC scores are reported in Fig. 2. Note that the higher the AUC, the better the performance.

There are striking differences between the Uniform Weight and Degree Weight schemes on one side, and the Mean Weight scheme on the other side. For the two former schemes, the results on the CATH2833 dataset show two different behaviors depending on the *β* values: for very small *β* values (below 10^−2^), all the AUC=f(*k*_*max*_) curves show an increase in performance, with relatively high values close to 0.75, while for larger values of *β* (>0.1), the same curves show a second increase in performance with different maxima for different *k*_*max*_ values, with the *β* values corresponding to these maxima decreasing as *k*_*max*_ increases. For the latter, however, the results are very different: while the same behavior is observed for the large values of *β*, poor fold recognition is observed for very small *β* values. As the two schemes Uniform Weight and Degree Weight are independent of protein length while Mean Weight depends on length, the discrepancy in behavior hints to WSeqKernel being able to pick differences in protein lengths for small *β* values under the first two schemes, as already reported for the unweighted kernel SeqKernel [29]. Using the Mean Weight scheme, however, the weighted kernel *K*_3_(*S,T*) associated to the matrix of ones is equal to 1, independent of the two proteins considered, leading to random behavior for fold recognition, and an AUC of 0.5. While interesting observations by themselves, we note that a string kernel with the Uniform or Degree weight scheme and a small value of *β* is not the type of string kernel we are interested in, as such a kernel is nearly independent of the actual sequences themselves, and mostly captures length differences. These results suggest to use the Mean Weight scheme instead, with large values of *β*. Figure 2 indicates that any value of *k*_*max*_ is possible, pending that the proper value for *β* is chosen. We suggest using the pair (*β*,*k*_*max*_)=(0.2,10), similar to our original suggestion for the unweighted kernel [29].

**Fig. 3:**
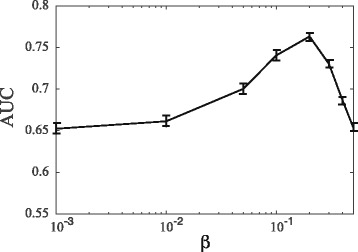
Statistical differences for WSeqKernel on different data sets. The mean performance over 10 randomized sets of proteins similar to CATH2833 the weighted string kernel WSeqKernel with the Mean Weight scheme and *k*_*max*_ set to 10, as measured by AUC, is plotted against the value of the *β* parameter. Error bars correspond to ± one standard deviation over these ten sets. Note the similarity with the corresponding curve for CATH2833 (see Fig. 2), with the same maxima around *β*=0.2

To reduce the risk that these observations are valid only to the specific proteins included in CATH2833, we repeated the process of generating CATH2833 with different initial random ordering of the proteins in CATH40e4 and generated ten independent CATH2833-like sets. The average overlap (i.e. percentage of shared proteins) between any of these sets and CATH2833 is 28%. For each set, we computed a curve *AUC*=*f*(*β*) under the Mean Scheme, with *k*_*max*_ set to 10. Results are shown in Fig. 3. The differences over the ten sets are very small, and not significant. Similar results were obtained with the two other weighting schemes (results not shown).

### WSeqKernel vs FASTA

With the exception of the length difference artifact, we observed that WSeqKernel performs best for fold recognition using the Mean Weight Scheme, *k*_*max*_=10, and *β*=0.2. With those parameters, the ROC analysis of the performance of WSeqKernel in detecting structural similarity as defined by CATH leads to an AUC of 0.69 for CATH2833. We repeated the same ROC analysis on CATH2833 using FASTA [39] for pairwise sequence comparison, and STRUCTAL [37] for 3D structure comparison. FASTA SSEARCH tool [39] implements a fast Smith and Waterman sequence comparison; the similarity is given either as a raw score, or as an E-value; we use the latter as a similarity measure. The ROC curve for the FASTA measure are marginally above random behavior, with an AUC score of 0.54 for CATH2833. This is expected, as by construction all protein pairs in those datasets have little or no sequence similarity. Assignment of structural fold is expected to work best when it is based on 3D structural information. Indeed, the AUC of 0.93 obtained based on the SAS STRUCTAL scores [37] illustrates excellent classification results. We note that even with X-ray structure information the classification is not perfect. It is possible that a small fully *α* or fully *β* protein is found to be similar to an *α*/*β* protein, based on local alignment of the helical or strand regions of the proteins. That said, STRUCTAL scores based on X-ray structures still perform remarkably well.

**Fig. 4:**
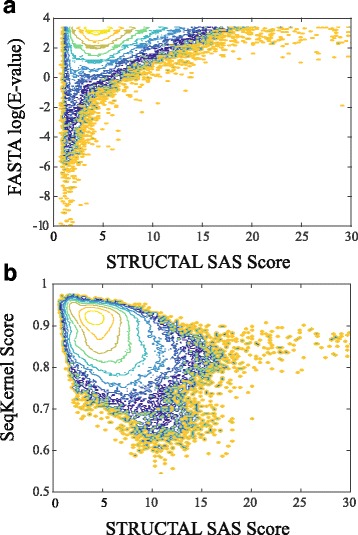
Probability–density distributions for protein comparison scores *S* contoured against SAS, the STRUCTAL alignment score along the horizontal axis and either ln(E-value) for the SSEARCH E-values (panel **a**), or the WSeqKernel similarity value (panel **b**) along the vertical axis. The densities are obtained by counting the number of pairs with particular SAS, E-values, and WSeqKernel values. Because of the wide range of density values, contours of *log*(*S*) are drawn with an interval of 1 (a full order of magnitude). Zero value are set to 0.001

In Fig. 4, we compared the SSEARCH E-values, the STRUCTAL SAS scores, and the kernel values computed with WSeqKernel for all pairs of proteins that belong to the same folds. We find that all protein pairs whose sequence alignments have low E-values have in parallel low SAS scores. The inverse, however, is not true: many protein pairs with low SAS scores, i.e. that are structurally similar, have high E-values, i.e. are not detected to be similar by SSEACH. Again, this is not unexpected as CATH2833 was specifically design to have this property. In contrast, there is a higher correlation (Pearson’s correlation coefficient of -0.42) between the WSeqKernel similarity score and the SAS score (Fig. 4 b). This result supports the idea that WSeqKernel recovers more structural information about the proteins in CATH2833 than FASTA.

### Amino acid properties recovered from protein structures

We can interpret Fig. 4 b as follows: given a substitution matrix, an optimized sequence comparison method recovers structure similarity scores relatively well, enabling reasonable fold recognition. This leads, however, to an interesting inverse problem: can we identify a substitution matrix that provides the maximum similarity between sequence comparison scores and structure comparison scores, and what is this optimized substitution matrix telling us about amino acids? As the equations defining the weighted string kernel provide analytical expressions for the sequence similarity score with respect to the scores for amino acid comparisons, we are in the right conditions to answer this question.

We first extracted all proteins from CATH2833 whose lengths are between 120 and 180 residues (793 proteins). By keeping the range of protein lengths small, we increase the impact of amino acid similarity on the string kernel. We performed an all-against-all comparison of the structures of those proteins using STRUCTAL [37]. We selected randomly a subset of pairs of proteins in this dataset such that the SAS score of their structural alignment is between 0 and 20, providing a uniform coverage of that range. This set includes 13177 pairs. The objective of the optimization procedure is to maximize the Pearson’s correlation coefficient between those SAS scores, and the corresponding kernel values obtained with WSeqKernel when comparing their sequences. The parameters that are optimized are the values in the upper triangle part of the matrix *K*_1_. We used the identity matrix as starting point of the optimization. This matrix includes no information on amino acid similarity. We note that the scores computed from *K*_1_ remain consistent with a kernel if and only if this matrix is maintained positive definite during the optimization.

Two sets of optimizations were performed using the Mean Weight scheme with two values of *k*_*max*_, namely 2 and 10, respectively. Both optimizations significantly improve the correlations between structural comparison scores and sequence comparison scores, from -034 to -0.63 for *k*_*max*_=2, and from -0.42 to -0.67 for *k*_*max*_=10. Interestingly, the optimization leads to increased values for the WSeqKernel scores compared to those computed with *K*_1_ set to the identity matrix. We note that the latter matrix is non-informative on similarities between amino acids of different types. It is inclined to favor perfect matches between k-mers. The correlations between the WSeqKernel scores and the SAS Structal scores obtained with this matrix are non significant. There are large ranges of WSeqKernel values for each SAS value. Those ranges are significantly reduced through the process of optimization, both for *k*_*max*_=2 and for *k*_*max*_=10 (see Additional file 1: Figure S1).

**Fig. 5:**
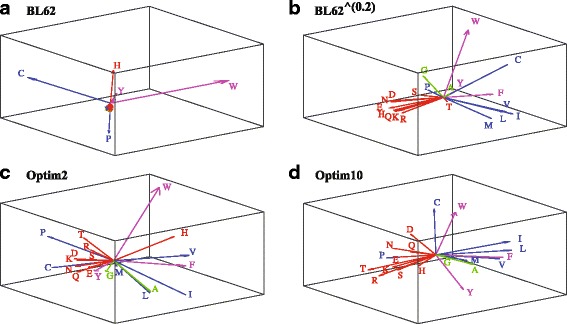
3D vector representations of amino acids as derived from the kernel matrices *K*_1_=*BL*62 and *K*_1_=*BL*62^*β*^ with *β*=0.2 (panels **a** and **b**, respectively), and from the optimized kernel matrices *K*_1_ for *k*_*max*_=2 and *k*_*max*_=10, (panels **c** and **d**, respectively). The proximity of these vectors relate to the chemical similarities of the amino acids they represent. To highlight this fact, we show the known polar amino acids (Q, R, E, K, N, D, T, H, and S) in red, the hydrophobic amino acids (M, V, L, I, P, and C) in blue, and the aromatic amino acids (Y, F, and W) in magenta. Note that the two small amino acids, A and G (shown in green), stand out

We use a graphical representation to highlight the information content of the corresponding optimized matrices, derived from Principal Component Analyses (PCA) of those matrices (see Additional file 1 for details). The principal components of a matrix identified by PCA correspond to linearly uncorrelated variables that best explain the data it contains. Once the principal components of a matrix *K*_1_ are known, amino acids are assigned “coordinates” along these components. In Fig. 5, we show the corresponding vectors in three dimensions for the un-optimized BL62, and BL62 ^0.2^ *K*_1_ matrices (where the exponent corresponds to the power of the matrix entries, not of the matrix), as well as for the two optimized matrices Optim2 and Optim10 corresponding to *k*_*max*_= 2 and 10, respectively. The choice of three dimensions is justified by considering the “energy” partition along the principal components. The first three components of the matrices Optim2 and Optim10 explain 87 and 95% of the energy of the matrix, respectively. For BL62, the first three components only explain 75% of the variance, and for BL62 ^0.2^, this number goes down to 63%. For those two matrices, a higher dimension would have probably been better. We kept it to three, to maintain the ability to plot the data and for comparison with the Optim matrices.

There is a striking difference between the two graphical representations of the matrices BL62 and BL62 ^0.2^. While the former shows a regrouping of most amino acids, with the exception of W, P, C, H, and Y, the latter shows a better spread of the amino acids, with groups that match with the physico-chemical properties of the amino acids. All the hydrophilic amino acids (in red) appear together, well separated from the hydrophobic amino acids (in blue). This difference validates the use of a *β* value to scale the input substitution matrix, and hints to using values for *β* below one, probably close to 0.1–0.2, in agreement with the parametrization results described above. The most striking result, however, is that the optimized *K*_1_ matrices Optim2 and Optim10 delineate amino acid similarities that are consistent with those observed in BL62 ^0.2^. Recall that those matrices were optimized against structural similarity scores, using the identity matrix as input to the optimization. The separation of hydrophilic versus hydrophobic amino acids is clearer in Optim10. Note that the optimized matrix Optim10 features W and C as being different. Cysteine can form disulphide bridges, while tryptophan is a large aromatic amino acid; both impose geometric constraints on protein structures. The differences between Optim2 and Optim10 also reinforce that a higher *k*_*max*_ value is preferred for applications of WSeqKernel to structure recognition.

The graphical representation of the substitution matrices shown above are informative on the similarities between amino acids that they capture. This information, however, is purely visual, therefore qualitative. We note that it is difficult to interpret the meaning of the principal components derived from a PCA analysis, as these are mathematically constructed to provide sub-components of the matrices with decreasing energy/information content. In order to provide a quantitative assessment of these principal components, we compared those components with the 544 amino acid indices available in the AAIndex database [41–43]. We performed this analysis on both BL62 ^0.2^ and Optim10, as those two matrices appear the most informative in Fig. 5.

For the first component of BL62 ^0.2^ that represents 33% of its variance, five of the 544 indices were selected with a correlation coefficient greater than or equal to 0.94: the “buriability” of Zhou and Zhou [44], an interactivity scale designed to correlate with hydropathy [45], a stability scale also from Zhou and Zhou, an hydrophobic parameter derived from free energy values for the transfer of amino acids to hydrophobic environment [46], and a normalized hydrophobicity scale [47]. Note that all these indices are related to amino acid burial and their hydrophobicity. These results are in agreement with the original findings of French and Robson [48], Swanson [49], Tomii and Kanehisa [42], and Gu et al. [50]. The best correlations between the second and third components of the matrix BL62 ^0.2^ with the amino acid indices contained in AAIndex are 0.77 and 0.70, respectively. The second component is found to correlate well with an amphiphilicity index, i.e. an index that characterizes amino acid preference at membrane-water interface [51], while the third component relates to statistics on turns in proteins [52].

Very similar results are found for Optim10. Its first component, which represents 73% of its variance, is found to correlate best with an accessibility reduction scale [53] and a normalized hydrophobicity scale [47]. Six of the top ten amino acid indices that best correlate with the first PCA component are shared between BL62 ^0.2^ and Optim10. All those ten indices relate to hydrophobicity. The second component for Optim10, which represents 19% of the variance, is found to correlate best with amino acid preferences at the C-termini of alpha-helices [54]. The third component, which only represents 2% of the variance, correlates with the third “principal property” of amino acids that was derived by Wald et al. [55] using PCA on twenty physico-chemical properties of amino acids.

Interestingly, the behavior observed for the first components differs from the results described by Kinjo and Nishikawa [56], who performed spectral analysis on substitution matrices compiled from protein structure alignments, including proteins with varying levels of sequence similarities. Using the same AAIndex database that we used [43], Kinjo and Nishikawa showed that at high sequence identities hydrophobicity plays a minor role, and that the “relative mutabilities” of Dayhoff [4] and Jones et al. [57] dominates. The difference between our results and those of Kinjo and Nishikawa is unclear.

### Improved fold recognition with the optimized kernel *K*_1_

**Fig. 6:**
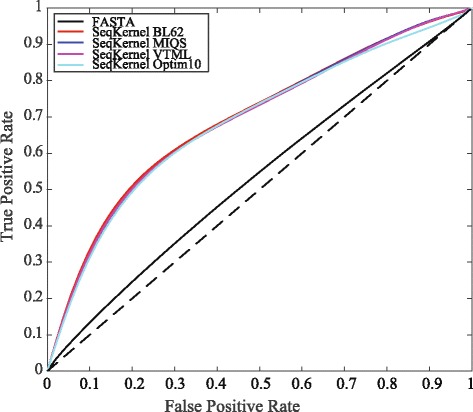
Choosing the substitution matrix for fold recognition. We compare the efficiency of FASTA pairwise sequence alignment method SSEARCH (black), with the weighted string kernel WSeqKernel method with four different input *K*_1_ matrices, (BL62) (red), Optim10 (cyan), MIQS (blue), and VTML (magenta) to detect fold similarity. “True” relationships are defined according to CATH topologies. The analysis is performed on CATH40e4 that contains 10619 sequences corresponding to 1363 folds (see Methods for details)

Our aim in optimizing the input amino acid kernel *K*1 for WSeqKernel starting from the identity matrix was to extract information on amino acids from protein structures. We checked if the resulting optimized matrix led to improved fold recognition. We performed ROC analyses of protein fold recognition based on SSEARCH E-values for pairwise sequence comparison, and on four conditions for WSeqKernel, with the input matrices BL62, Optim10, MIQS, and VTML, respectively. VTML was selected as it provides improved global pair-wise alignment on existing protein sequence alignment benchmarks [58], while MIQS was developed as an improved substitution matrix for fold recognition [59]. For all WSeqKernel runs we set the parameters *β* and *k*_*max*_ set to 0.2 and 10, respectively, and use the Mean Weight scheme. Results are shown in Fig. 6.

We find that all the ROC curves based on WSeqKernel are significantly better than the ROC curve for the FASTA score. The AUC for the FASTA curve is 0.53, while the AUCs for WSeqKernel with BL62, MIQS, VTML, and Optim10 are all equal to 0.69. It is worth noticing that there are not noticeable differences between the performances of those four substitution matrices, at least on the database we considered, Cath40e4.

### Comparisons with other string kernels

**Fig. 7:**
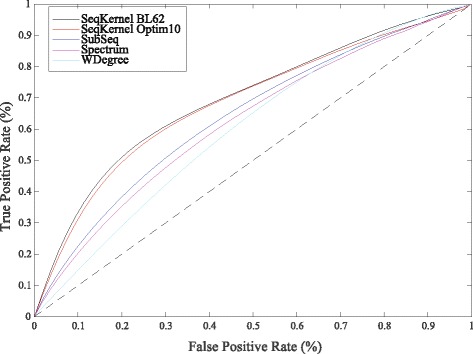
The weighted string kernel WSeqKernel versus other string kernel methods. We compare the performance of five different distances between protein sequences for detecting remote homologies. Those distances include the distances based on the weighted string kernel defined in this work, with (*β*,*k*_*max*_)=(0.2,10), the weights set according to the Mean Weight scheme, and the BL62 substitution matrix and Optim10 substitution matrices, and three other string kernel distances, Subseq [23], Spectrum [24], and WDegree, a weighted string kernel with different weights [25, 26]) “True” relationships are defined according to CATH topologies. The analysis is performed on CATH40e4 that contains 10619 sequences corresponding to 1363 folds

We repeated the ROC analyses presented above on the full Cath40e4 database using five possible distances between protein sequences, all derived from string kernels. The first and second distances correspond to the weighted kernel WSeqKernel with the parameters *β* and *k*_*max*_ set to 0.2 and 10, respectively, with the Mean Weight scheme, and with BL62 and Optim10 as substitution matrices, respectively. The other three distances between sequences are derived from other string kernels. We included the subsequence string kernel introduced by Lodhi et al. [23], Subseq, the Spectrum string kernel originally proposed by Leslie et al. [24], and the weighted string kernel WDegree of Rätsch and colleagues [25, 26]. For those last kernel-based distances between sequences, we used the package Harry [60, 61], with all parameters set to their default values. Results of the classification experiments are given in Fig. 7.

The classifications obtained with WSeqKernel with the Mean Weight Scheme and (*β*,*k*_*max*_) set to (0.2,10) are similar to each other, with AUC values of 0.69, and significantly more accurate than those observed with the other three sequence-based distances, whose AUC values are 0.63, 0.62, and 0.60 for Subseq, Spectrum, and WDegree, respectively. We note that the latter approaches, while they resemble the WSeqKernel method, do not consider similarities between amino acids, the way the *K*_1_ kernel does; this kernel is at the core of WSeqKernel.

## Discussion

This paper draws from the concept of string kernels applied to biological sequence analysis [12–14]. It describes an alignment-free method for protein sequence comparison that is based on a modified version of the string kernel introduced by Smale and collaborators [28]. In contrast with the previous studies on string kernels, we do not include at this stage our kernel into any learning algorithms. Instead, we assess directly its ability to classify proteins into structural folds based on sequence information only. We note that our string kernel, WSeqKernel, relies on two options, namely the choice of the weighting scheme that modulates the impact of the lengths of the sequence substring considered, and the choice of the substitution matrix that is used to score matches of pairs of amino acids, as well as on two parameters, *β* that modulates the input substitution matrix, and *k*_*max*_ that defines the maximum size of the k-mers that are considered. We provide an exhaustive analysis of the effects of these two options and of the two parameters on the performance of the kernel for fold recognition. Such an analysis, which is necessary as a first step to improving string kernel methods, was only partially included in the presentations of the equivalent kernels defined by Saigo et al. [27], and by Smale and co-workers [28]. It is the first focus of this paper. Using a dataset that only contains remote homologs, we have shown that WSeqKernel performs remarkably well for small values of *β* (<10^−2^) with a uniform weighting scheme, independently of the choice for *k*_*max*_. This behavior is similar to the results obtained with SeqKernel, the unweighted version of the string kernel [29]. With such small values of *β*, however, SeqKernel is tuned to capture the difference in lengths of the protein sequences being compared, which is not of interest for fold recognition [29]. Using a weighting scheme that averages out the contributions of all k-mers, we have shown that we can significantly reduce this behavior. We have shown that for larger values of *β*, there are pairs of values (*β*, *k*_*max*_) that provide significant performance in fold recognition. We suggest to use the pair (*β*,*k*_*max*_)=(0.2,10).

WSeqKernel, just like any alignment-based sequence comparison method, depends on a substitution matrix. Such a matrix provides a quantitative measure of the similarities of amino acids. Inclusion of such information has already been shown to improve the power of string kernels applied to sequence analysis [28, 62]. The main contribution of this paper is to propose a framework for optimizing this matrix so that sequence similarity scores show improved correlation to structure similarity scores. Otherwise stated, we have reversed engineered the problem of protein fold recognition: given scores that provide good fold recognition, we have developed a mechanism that allows us to find which amino acid similarity matrix would enable a string kernel to mimic those scores. We have shown that starting from no information on similarity between different amino acid types, we were able to derive with this framework a kernel matrix Optim10 that captures similarities between amino acids reflecting their physico-chemical properties. That we are able to retrieve this information from Optim10 reinforces the idea that it is possible to extract the sequence information embedded in protein structures.

We have tested different substitution matrices as input to WSeqKernel: the well-recognized BLOSUM 62 matrix that is used by default by many sequence comparison methods, recently optimized matrices such as VTML [58] and MIQS [59], and our own optimized matrix Optim10. We observed similar results in terms of fold recognition on a large database of non-redundant protein sequences, Cath40e4. The lack of differences can be assigned to two possible reasons. First, WSeqKernel itself may be insensitive to the input substitution matrix. We did observe, however, that WSeqKernel performs better than other string kernel methods that do not consider relative similarities between amino acids (see Fig. 7). A second possibility is that all the matrices we have considered are not significantly dissimilar to each other. This is for example the case for BL62 and Optim10, as illustrated in Fig. 5. We are currently testing more amino acid substitution matrices to confirm that the second reason is valid. We do believe that there is still room for improvement when designing substitution matrices.

We have proposed a simple algorithm for computing the kernel score between two sequences **S** and **T**. The time complexity of this algorithm is *O*(*nmk*_*max*_), where *n* and *m* are the lengths of the sequences **S** and **T**, respectively, and *k*_*max*_ is the maximum length of the k-mers considered. Even if *k*_*max*_ is set to a small value (such as the value of ten suggested in this paper), this computational cost remains high as it is of the order of the square of the protein sequence lengths. This should be compared to the computing time of other approaches for alignment-free sequence comparisons that are based on frequencies of occurence of k-mers. Those methods have linear time complexities with respect to sequence length, which makes them amenable to whole genome sequence comparison (for review, see for example Song et al. [63]). However, we see those methods and WSeqkernel as serving different purposes. While the former provides fast filtering when comparing a large number of sequences, or very large sequences, WSeqKernel provides a rigorous mathematical framework for comparing sequences using an exact metric. We acknowledge, however, that WSeqKernel comes with a high computational cost. We are currently looking at possibilities to reduce this cost, by considering for example random selections of k-mers.

## Conclusions

This paper represents work in progress. We do not claim that we have designed a string kernel that can solve the fold recognition problem. We have made progress in characterizing and parametrizing such string kernels, and we have shown that they provide a framework for extracting sequence information from structure. There are many open questions, however, that need to be addressed. String kernels do provide a mathematical framework for comparing protein sequences. They assume independence of neighbor amino acids, an hypothesis whose impact needs to be tested. It is unclear whether the weighting scheme proposed in this paper is optimal. More generally, it remains to be seen if additional information can be incorporated in those kernels. We are also interested in extending the applications of such kernels to study 3D structures of proteins. We intend to address these questions in future studies.

## Additional file

Additional file 1 Method: Optimization of an amino acid substitution matrix. Comprehensive description of the method for optimizing the substitution matrix given as input to WSeqKernel. (PDF 809 kb)
