# Supplementary material for: A weighted string kernel for protein fold recognition
Source: BMC Bioinformatics. 2017 Aug 25;18:378. doi: 10.1186/s12859-017-1795-5 (PMC5574112; doi:10.1186/s12859-017-1795-5)
Supplement: Additional file 1 — Method: Optimization of an amino acid substitution matrix. Comprehensive description of the method for optimizing the substitution matrix given as input to WSeqKernel. (PDF 809 kb) [file 12859_2017_1795_MOESM1_ESM.pdf]

Optimizing amino acid substitution matrices for protein fold recognition

Supplementary Material

Saghi Nojoomi and Patrice Koehl

April 5, 2017

Methods

Optimization of the amino acid kernel K_1

Here we re-introduce the procedure for optimizing the input substitution matrix K_1 for the string kernel SeqKernel defined in the main body of the paper. We focus on the technical details of this procedure, namely we describe the calculations of the derivatives of the objective function as well as the algorithm used to perform the optimization.

Objective function. Let $\mathcal{L}$ be a set of pairs of proteins. We note N the cardinality of $\mathcal{L}$. For each pair n of proteins in $\mathcal{L}$, we compute the alignment between their structures using STRUCTAL [1], and record its SAS score, Y_n. This score is an input to the procedure. In parallel, we compute the kernel between their sequences, using SeqKernel, and record it as X_n. X_i is a non-linear function of the elements of the kernel K_1. Our objective is to optimize the degree of linear dependence between the two variables Y and X. We quantify this linear dependence using the Pearson's correlation coefficient, which we denote

as P:

$$P = \frac{N\sum_{n=1}^{N} Y_n X_n - \sum_{n=1}^{N} Y_n \sum_{n=1}^{N} X_n}{\sqrt{N\sum_{n=1}^{N} Y_n^2 - \left(\sum_{n=1}^{N} Y_n\right)^2}\sqrt{N\sum_{n=1}^{N} X_n^2 - \left(\sum_{n=1}^{N} X_n\right)^2}} \quad \text{(S1)}$$

P takes value between -1 and 1.

Derivatives of the objective function. The kernel matrix K_1 is a 20×20 symmetric, positive, definite matrix. As such, it contains 210 independent variables. Let $K_1(i,j)$ be one of those variables, where $i \in [1,20]$ and $j \in [i,20]$. We compute the derivative of the objective function P with respect to $K_1(i,j)$ using simple rules for computing derivatives. Let us rewrite first $P = P_1/P_2$, then

$$\frac{\delta P}{\delta K_1(i,j)} = \frac{1}{P_2}\frac{\delta P_1}{\delta K_1(i,j)} - \frac{P_1}{P_2^2}\frac{\delta P_2}{\delta K_1(i,j)} \quad \text{(S2)}$$

where:

$$\begin{aligned}
\frac{\delta P_1}{\delta K_1(i,j)} &= N\sum_{n=1}^{N} Y_n \frac{\delta X_n}{\delta K_1(i,j)} - \sum_{n=1}^{N} Y_n \sum_{n=1}^{N} \frac{\delta X_n}{\delta K_1(i,j)} \\
\frac{\delta P_2}{\delta K_1(i,j)} &= \frac{\sqrt{N\sum_{n=1}^{N} Y_i^2 - \left(\sum_{n=1}^{N} Y_i\right)^2}}{\sqrt{N\sum_{n=1}^{N} X_n^2 - \left(\sum_{n=1}^{N} X_n\right)}}\Bigg[2N\sum_{n=1}^{N} X_i \\
&\quad \frac{\delta X_n}{\delta K_1(i,j)} - 2\left(\sum_{n=1}^{N} X_n\right)\sum_{n=1}^{N}\frac{\delta X_n}{\delta K_1(i,j)}\Bigg] \quad \text{(S3)}
\end{aligned}$$

We therefore need to compute the $\frac{\delta X_n}{\delta K_1(i,j)}$ for all pairs of proteins n in $\mathcal{L}$.

Let S and T be the two sequences corresponding to the pair n. Then $X_n = \hat{K}_3(S,T)$, and:

$$\begin{aligned}
\frac{\delta X_n}{\delta K_1(i,j)} &= \frac{1}{\sqrt{K_3(S,S)K_3(T,T)}}\frac{\delta K_3(S,T)}{\delta K_1(i,j)} - \frac{\hat{K}_3(S,T)}{2} \\
&\quad \left(\frac{1}{K_3(S,S)}\frac{\delta K_3(S,S)}{\delta K_1(i,j)} + \frac{1}{K_3(T,T)}\frac{\delta K_3(T,T)}{\delta K_1(i,j)}\right)
\end{aligned}$$

(S4)

For any sequences S and T, $K_3(S,T)$ is a weighted sum of kernels $K_3^k(S,T)$, where k is the length of the k-mers considered. $K_3^k(S,T)$ is itself a sum of kernels K_2^k computed all all pairs of k-mers of length k in S and T. Let u^k and v^k be two such k-mers. Then,

$$\frac{\delta K_2^k(u^k, v^k)}{\delta K_1(i,j)} = \sum_{l=1}^{k} \prod_{m=1, m \neq l}^{k} K_1(u_m, v_m) \delta[(u_l, v_l), (i,j)] \tag{S5}$$

where $\delta[(u_l, v_l), (i,j)] = 1$ if $(u_l, v_l) = (i,j)$ or $(u_l, v_l) = (j,i)$, and 0 otherwise. The terms $\frac{\delta X_n}{\delta K_1(i,j)}$ are then computed by summing all derivatives given by Equation S5, for all k-mers of size k in S and T, and for all $k \in [1, k_{max}]$.

Maintaining the kernel K_1 positive definite. Direct minimization of the objective function defined by Equation S1 is likely to fail as there are no guarantee that the matrix K_1 stays positive definite. One option to circumvent this problem is to consider the Cholesky factorization of K_1. Indeed, any positive definitive real matrix K_1 can be decomposed as

$$K_1 = LL^T \tag{S6}$$

where L is a lower triangle matrix. Conversely, if K_1 is a matrix that can be written as LL^T for some invertible lower triangular matrix L, then K_1 is positive definite. The latter provides a framework for enforcing positive definiteness, namely we set the parameters of the optimization to be the matrix L, the Cholesky factorization of the kernel K_1. We do need to impose that the matrix L remains invertible, which is achieved by preventing any of the coefficients $L(i,i)$ to become zero. The change of variables from K_1 to L is fully defined once we have a means to compute one set from the others, and once we can transfer derivatives. The former is given by Equation S6. In practice, we only compute the factorization once; this factorization is in fact trivial, as our input kernel K_1 is the identity matrix and the corresponding L is also the identity matrix. The latter is given by the chain rule. Notice first that:

$$K_1(i,j) = \sum_{k=\min(i,j)}^{20} L(i,k)L(j,k) \tag{S7}$$

Second, we apply the chain rule,

$$\frac{\delta P}{\delta L(i,j)} = \sum_{(k,l)} \frac{\delta P}{\delta K_1(k,l)} \frac{\delta K_1(k,l)}{\delta L(i,j)} \tag{S8}$$

where the derivatives $\frac{\delta K_1(k,l)}{\delta L(i,j)}$ are directly computed from Equation S7.

An algorithm to optimize the kernel K_1. Combination of all the elements described above (i.e. the choice of the objective function, computation of its derivatives, and enforcement of positive definiteness of the kernel matrix) leads to algorithm 1 for optimizing K_1.

Algorithm 1 *OptimK1, an algorithm for refining a substitution matrix based on structure comparison data*

Input: $\mathcal{L}$, list of pairs of proteins; Y_n, corresponding SAS values; k_{max}, largest k-mer considered

(1) **Initialize:** Set $K_1^0 = I$ (the identity matrix); compute L^0, the Cholesky factorization of K_1^0.

for $n = 1, \ldots$ until convergence **do**

(2) Compute $K_1^n = L^{n-1}[L^{n-1}]^T$

(3) For all n in $\mathcal{L}$, compute X_n and $\frac{\delta X_n}{\delta K_1^n(i,j)}$, the kernel value based on K_1^n and its derivatives with respect to the elements of K_1^n.

(4) Compute the objective function P^n using equation S1 and its derivatives with respect to the elements of K_1^n

(5) Convert derivatives with respect to the elements of K_1^n to derivatives with respect to elements of L^{n-1} using Equation S8

(6) Check for convergence: if $|P^n - P^{n-1}| < TOL$ stop

(7) Update $L^{n-1} \rightarrow L^n$

end for

Output: the optimized matrix K_1^{opt}, the corresponding kernel values X for protein pairs in $\mathcal{L}$, and the correlation coefficient P.

The time consuming part of this algorithm is concerned with computing all kernel values and their derivatives for the protein pairs in $\mathcal{L}$ (step 3). We note however that this part of the algorithm is embarrassingly parallelizable. The Cholesky factorization in step

(1) is trivial when K_1^0 is the identity matrix I_{20}, in which case L^0 is the same identity matrix. For a more general starting matrix K_1^0, we have used a C++ version of the Cholesky decomposition code proposed by Healy [2]. The optimization itself is iterative. There are many ways in which the parameters L^{n-1} can be updated in step (7), such as steepest descent or conjugate gradients, or even second order Hessian-based method such as Newton's method. We have used an intermediate between those two approaches, L-BFGS-B [3]. This method builds an estimate of the Hessian as it proceeds along the optimization, saving the time that the computation of the Hessian would require. It converges faster than steepest descent or conjugate gradients. This version also considers bounds on the variables, which allowed us to enforce that the elements of the diagonal of the matrix L remains strictly positive.

Supplemental Results

Optimizing the input amino acid kernel K_1

We extracted 793 proteins from CATH2833 whose lengths are between 120 and 180 residues. We performed an all-against-all comparisons of the structures of those proteins using STRUCTAL [1]. We selected randomly a subset of 13177 pairs of proteins in this dataset such that the SAS score of their structural alignment is between 0 and 20. The objective of the optimization procedure is to find a kernel matrix K_1 that maximizes the Pearson's correlation coefficient between those SAS scores, and the corresponding kernel values obtained with SerKernel (see Equation S1). We used the identity matrix as starting point of the optimization. Two sets of optimizations were performed, with two values of k_{max}, namely 2 and 10, respectively. Results are presented in Figure S1.

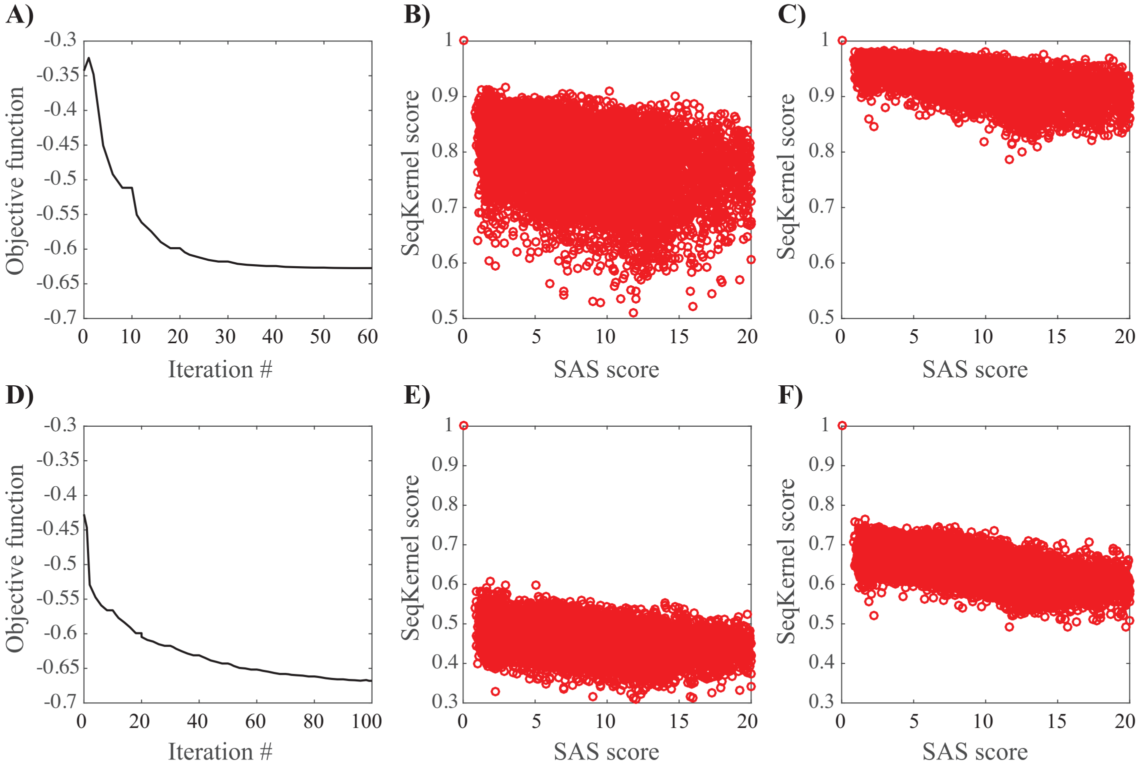

Figure S1: **Optimizing the input kernel K_1 for SeqKernel.** The matrix K_1 is optimized to maximize the Pearson's correlation coefficient P between STRUCTAL SAS scores, and the corresponding kernel values for 13177 pairs of proteins from CATH2833 (see text for details). Two versions of SeqKernel are considered, with the maximum size k_{max} of the k-mers included in the calculation of the kernel set to 2 and 10, respectively. Results for $k_{max} = 2$ are shown in panels A, B, and C, while the equivalent results for $k_{max} = 10$ are shown in panels D, E, and F. In panels A and D, P is plotted against the iteration number during the optimization. The Seqkernel scores prior to (panels B and E), and after optimization (panels C and F) are plotted against the STRUCTAL scores.

References

[1] S. Subbiah, D.V. Laurents, and M. Levitt. Structural similarity of DNA-binding domains of bacteriophage repressors and the globin fold. *Curr. Biol.*, 3:141–148, 1993.

[2] M. Healy. Algorithm AS 6: triangular decomposition of a symmetric matrix. *Applied Statistics*, 17:195–197, 1968.

[3] C. Zhu, R.H. Byrd, P. Lu, and J. Nocedal. Algorithm 778: L-BFGS-B: Fortran subroutines for large-scale bound-constrained optimization. *ACM Trans. Math. Soft.*, 23: 550–560, 1997.
